# Rare Strain of *Vibrio cholerae* Septicemia in a Patient with Multiple Myeloma

**DOI:** 10.1155/2015/596906

**Published:** 2015-07-15

**Authors:** Deepu Daniel, Sunil Kumar

**Affiliations:** ^1^Broward Health Medical Center, Fort Lauderdale, FL 33316, USA; ^2^Pulmonary/Critical Care Medicine, Broward Health Medical Center, Fort Lauderdale, FL 33316, USA

## Abstract

*Introduction*. Non-O1/non-O139 is a rare strain of *Vibrio cholera* that has been documented to cause significant morbidity and mortality in the immunosuppressed population. *Case Presentation*. A patient with multiple myeloma develops non-O1/non-O139 *Vibrio cholera* septicemia, leading to multiorgan failure and ultimately death. *Discussion*. An exceedingly rare strain of *Vibrio cholera*, non-O1/non-O139, may be an important factor of morbidity and mortality in certain immunosuppressed populations, such as patients with multiple myeloma and malignancies. *Conclusion*. Bacteremia involving generally noninvasive microbes, such as non-O1/non-O139 *Vibrio cholerae*, can have significant deleterious effects in the immunosuppressed patients as shown by this case report. Physicians need to be more diligent when treating these patients.

## 1. Introduction

Reported cases of non-O1/non-O139* Vibrio cholerae* bacteremia are extremely uncommon in the literature [1]. This strain has, on rare occasions, been shown to cause invasive and systemic manifestations beyond the commonly associated gastrointestinal symptoms of diarrhea, nausea, and emesis [2–4]. Bacterial infections, including such rare instances of non-O1/non-O139* Vibrio cholerae*, are more prone to occur in the immunosuppressed and may carry worse prognoses in this population [5]. One such population of patients is those with multiple myeloma. This case study presents a peculiar incidence of severe sepsis induced by non-O1/non-O139* Vibrio cholerae* bacteremia in a multiple myeloma patient leading to severe sepsis, pulmonary hemorrhage, and ventilator dependent respiratory failure.

## 2. Case Presentation

A 54-year-old Haitian male with a past medical history of multiple myeloma diagnosed one year prior was admitted to Broward Health Medical Center due to septic shock and acute renal injury. He had his last chemotherapy session for the multiple myeloma over 3 months ago and was lost to follow-up since that time period. Symptomatically, he was complaining of diffuse abdominal pain, nausea, vomiting, and diarrhea for 3 days after returning from a trip to Haiti.

Initial vital signs included temperature of 102 degrees Fahrenheit, heart rate 90 beats per minute, blood pressure 86/40 mmHg, and oxygen saturation 100% on 2 L/min of oxygen via nasal cannula. Labs showed a white blood cell count of 2.85 × 10^3^/*μ*L, hemoglobin 8.7 g/dL, hematocrit 25.5%, platelets 43 × 10^3^/*μ*L, segmented neutrophils 76%, bands 8%, and lymphocytes 12%. Comprehensive metabolic panel showed sodium 144 mmol/L, potassium 3.9 mmol/L, chloride 119 mmol/L, bicarbonate 12 mmol/L, BUN 31 mg/dL, creatinine 3.5 mg/dL, alkaline phosphatase 39 units/L, aspartate aminotransferase 55 units/L, alanine aminotransferase 65 units/L, and albumin 2 g/dL. Initial X-ray and CT of the chest did not indicate any acute infiltrates. A CT of the abdomen showed mild circumferential wall thickening of the colon extending from the cecum to the rectum indicative of colitis. Aggressive IV fluid hydration was initiated and he was empirically started on piperacillin-tazobactam. Initial blood cultures preliminarily grew gram negative rods, at which time levofloxacin was added to his regimen.

Within 48 hours of admission he began to experience worsening respiratory distress and severe tachypnea, with ABG indices showing a pH of 7.26, pCO_2_ of 43, pO_2_ of 64, base excess of (−8), and O_2_ saturation of 91%. Patient was switched to a nonrebreather. He was found to be fluid overloaded with a significant positive fluid balance. The patient had to be placed on BiPap and diuresed with bumetanide. Repeat chest X-ray indicated new right upper lobe infiltrates. After being stabilized he was weaned back to 4 L of O_2_ via nasal cannula.

Blood culture identification showed growth of* Vibrio cholerae* non-O1/non-O139. Based on identification and susceptibility studies, antibiotic treatment was deescalated to intravenous levofloxacin. The bacterial strain was isolated and identified by the Florida Department of Health in Jacksonville, FL.

However, the patient's clinical status once again deteriorated. He began to develop episodes of copious hemoptysis and worsening thrombocytopenia. Intermittent platelet transfusions and a trial of desmopressin were started; however no significant increase in platelet count was seen. Patient's respiratory status continued to worsen through his course progressing to respiratory failure requiring emergent intubation. He continued to have bloody secretions suctioned from the endotracheal tube. Chest X-rays indicated worsening bilateral fluffy infiltrates. In the setting of continued bloody secretions it was felt that the radiological findings may correlate with pulmonary hemorrhage.

Patient was sent to the operating room for tracheostomy placement and oral packing by the otolaryngologist. Secondary to the extensive amount of oropharyngeal bleeding, vaginal pads were required. The patient was also started on aminocaproic acid, a fibrinolytic inhibitor without any significant improvement. He continued to require full ventilator support and was too hemodynamically unstable to attempt weaning from the ventilator. As his clinical status deteriorated further, he became less responsive and comatose. A CT of the brain revealed a 2 cm hemorrhage in the left cerebellum with mild surrounding edema. He later underwent cardiac arrest and was unable to be revived, ultimately succumbing to his illness.

## 3. Discussion

As mentioned earlier, immunosuppressive states such as multiple myeloma and other hematologic malignancies do place patients at an overall increased risk of bacteremia [7–12]. Hypogammaglobulinemia and a decrease in the production of IgA and IgG may lead to an overall increased risk of infection specifically among those with multiple myeloma. These patients are functionally asplenic and are thus susceptible to bacterial infections, particularly encapsulated microbes [13, 14]. However, a non-O1/non-O139* Vibrio cholerae* infection has rarely been documented. This case showed the deleterious effects of an exceedingly rare bacterium on an immunosuppressed host.

Shelton et al. described a case report of recurrent non-O1* Vibrio cholerae* bacteremia in a patient with multiple myeloma [15]. The patient had been admitted both instances, approximately one year apart, with symptoms of fever, malaise, and cough. During both instances, patient did not have any gastrointestinal complaints of diarrhea, nausea, or vomiting. Patient had also denied consumption of raw seafood. The blood cultures drawn during both admissions revealed infection with non-O1* Vibrio cholerae*. Comparison of the two strains of non-O1* Vibrio cholerae* showed minor differences to each other. Patient was discharged home both instances on oral antibiotic therapy.

Majority of pathogenic* Vibrio cholerae* strains express two “O antigens,” O1 and O139. Those that do not express these antigens are classified as non-O1/non-O139* Vibrio cholerae*. The majority of these latter strains are for the most part nonpathogenic. However there has been a minority that has caused significant pandemics and outbreaks [16–20]. Two main virulence factors that are found in O1 and O139* Vibrio cholerae* are the cholera toxin (CT) and toxin coregulated pilus (TCP) [21]. CT is responsible for causing diarrhea whereas TCP functions in allowing the microbe to colonize the colon [22]. However the mechanism of pathogenicity in non-O1/non-O139* Vibrio cholerae* is less well established.

Dziejman et al. performed genetic analyses on four strains of non-O1/non-O139* Vibrio cholerae* (AM-19226, AM-15622, MZO-2, and MZO-3) which were isolated from stool studies from patients in Bangladesh in 2001 [23]. Genomic analyses of the AM-19226 strain showed similarities to genes found in* Vibrio parahaemolyticus* that encode for the Type III Secretion System (TTSS). TTSS produces virulence factors in* V. parahaemolyticus*, as well as in several gram negative organisms (i.e.,* Salmonella, Shigella, Vibrio, E. coli, Pseudomonas, Aeromonas*). TTSS found in* Vibrio parahaemolyticus* functions in causing enterotoxicity. This study suggested the possibility that the TTSS gene cluster in AM-19226 would produce similar virulence factors and that similar TTSS clusters may lead to virulence factors in other non-O1/non-O139 strains.

**Figure 1:**
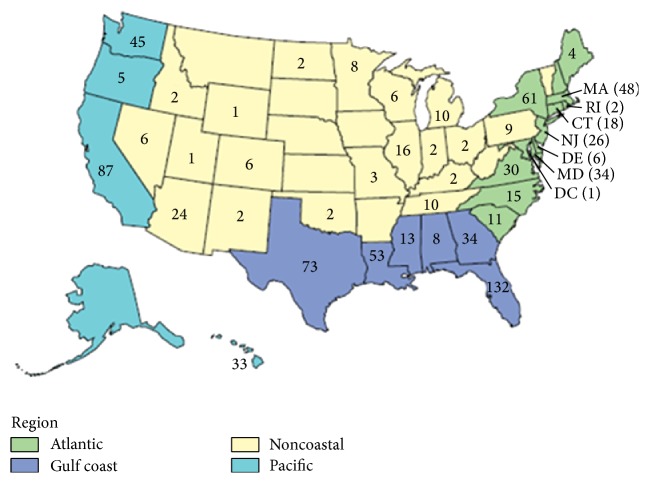
Geographic distribution of* Vibrio* infections in the United States in 2011, also taken directly from the 2011 COVIS report published on the CDC website [6].

The reported incidence of non-O1/non-O139 bacteremia is very rare in the United States. According to the CDC's 2011 COVIS (Cholera and Other* Vibrio* Illness Surveillance) annual summary, there were only 68 reported cases of bacteremia attributable to non-O1/non-O139* Vibrio cholerae* in the United States (Table 1 and Figure 1) [6].

One of the most significant cholera outbreaks ever to occur in the western hemisphere occurred in Haiti in 2010. The patient in this case had confirmed to hospital staff that he has been living in Haiti for nearly 6 months prior to his admission. Chin et al., implementing third generation single molecule DNA sequencing, analyzed the genetic sequences of two prominent* Vibrio cholerae* strains isolated during the Haiti outbreak and compared them to strains seen during earlier outbreaks in South Asia and Latin America [24]. Studies indicated that the likely causative strain of the Haiti outbreak was* Vibrio cholerae* El Tor. This was the same strain isolated from Bangladesh in 2002 and 2008.

This patient passed away from severe sepsis, respiratory failure, pulmonary hemorrhage and copious retropharyngeal bleeding exacerbated by unrelenting thrombocytopenia. Despite aggressive intravenous fluid hydration, appropriate antibiotic therapy, and multiple transfusions of packed red blood cells and platelets, his illness proved fatal. His underlying multiple myeloma was his most significant comorbidity contributing to his unfortunate outcome. Virulence factors produced by this rare strain of* Vibrio cholerae* may have further attributed to his prognosis; however we are unable to definitively make this conclusion at this time. Further studies will be required to identify and study the pathogenicity virulence factors associated with this microbe.

## 4. Conclusion

In our case report, we described a fatal case of severe sepsis in a patient with multiple myeloma. The patient's clinical course was complicated by his history of multiple myeloma. It is important to reemphasize the rarity of reported cases of bacteremia caused by these strains of bacteria and that the immunosuppressed population is more prone to bacterial infections, including such rare strains.

It is important for physicians to understand the deleterious effects that a generally nonpathogenic strain of bacteria can have on immunosuppressed patients. Physicians need to be vigilant on this particular population of patients to avoid these consequences. Our case was important in reiterating this fact.

## Figures and Tables

**Table 1:** Reported *Vibrio* cases in the United States in 2011, taken directly from the 2011 COVIS annual report published in the CDC website [6].

			Demographic characteristics				Outcomes			
*Vibrio* species	Cases		Age (years)		Sex		Hospitalizations		Deaths	
	*N*	%	Median	Range	Male (*n*/*N*)	%	*n*/*N*	%	*n*/*N*	%
*V. alginolyticus *	156	18	33	2–86	118/155	76	16/146	11	0/144	0
*V. cholerae* (excluding toxigenic O1 and O139)^*∗*^	86	10	48	1–85	59/86	69	28/82	34	3/80	4
*Photobacterium damselae* subsp. *damselae* (formerly *V. damselae*)	7	1	55	6–77	4/7	57	3/6	50	0/1	0
*V. fluvialis *	37	4	65	20–108	18/37	49	18/34	53	0/33	0
*Grimontia hollisae* (formerly *V. hollisae*)	7	1	50	42–75	7/7	100	4/7	57	0/6	0
*V. mimicus *	15	2	45	4–87	11/14	79	6/15	47	0/15	0
*V. parahaemolyticus *	334	39	45	1–94	225/334	67	75/315	24	7/304	2
*V. vulnificus *	113	13	60	8–91	87/111	78	89/113	87	34/108	31
Species not identified	87	10	44	3–93	51/86	59	19/82	23	4/78	5
Multiple species^†^	11	1	52	23–80	7/11	64	4/11	36	0/10	0
Total	**853**	**100**	**47**	**1–108**	**587/848**	**69**	**272/811**	**34**	**48/785**	**6**

^*∗*^Including 86 nontoxigenic *V. cholerae *(non-O1/non-O139 [68 cases], O1 [2 cases], O139 [1 case], and no serogroup specified [2 cases]) and 13 toxigenic *V. cholerae* (O75 [12 cases] and O141 [1 case]).

^†^The following combinations of *Vibrio* species were isolated from patients infected with multiple species: *V. alginolyticus, V. parahaemolyticus* (3 patients); *V. cholerae* O1, *V. parahaemolyticus* (1 patient); *V. fluvialis, V. parahaemolyticus* (1 patient); *P. damselae* subsp. *damselae, Vibrio* species not identified (1 patient); *V. fluvialis, V. furnissii* (1 patient); *V. parahaemolyticus*, *V. vulnificus* (1 patient); *V. cholerae* non-O1/non-O139, *Vibrio* species not identified (1 patient); *V. alginolyticus, Vibrio* species not identified (1 patient); *V. alginolyticus, P. damselae* subsp. *damselae* (1 patient). None of these are included in the rows for individual species.
